# Supplementary material for: “I was scared dating… who would take me with my status?”—Living with HIV in the era of UTT and U = U: A qualitative study in Johannesburg, South Africa
Source: PLOS Glob Public Health. 2023 Oct 13;3(10):e0000829. doi: 10.1371/journal.pgph.0000829 (PMC10575521; doi:10.1371/journal.pgph.0000829)
Supplement: S1 Table — (PDF) [file pgph.0000829.s001.pdf]

Code	Description	Example
**1.Meaning ascribed to HIV and PLHIV self-image in the UTT era**		
Increased normalization of HIV	HIV was seen as a “common”disease by most participants. And majority of the participants knew many people who were living with HIV.	“I can’t stop taking my medication because I am HIV positive, a lot of people are living normal lives, I’m not the only person and I’m not the first one.” – Female 23, key informant interview
Persistent HIV stigma is linked to sexual transmission	Despite the waning association of HIV with death, HIV still remained highly stigmatised as a sexually transmitted disease. Stigma is a “mark of disgrace”	"I started treatment in 2017 because I was in denial, was hiding it even at home, this thing and was scared to talk about this situation. Ok to think about it, the fact that I broke my virginity when I was 21 means I had to discipline myself, thinking that you know what, unfortunately at the time I was broke my virginity I got pregnant and I got HIV and it was so sad to me that I had disciplined myself for such a long time I was
**2. Challenges PLHIV experienced related to their HIV status regarding relationships and disclosure**		
Transmission Anxiety	Transmission-related HIV stigma had a profound impact on PLHIV’s expectations and experiences of sexual relationships. There is of fear transmitting HIV to their sexual partners, responsibility for avoiding transmission, and fears of being stigmatised by others for being a risk of transmitting HIV to other people	“The only thing that scares me is the possibility of transmitting this virus.”- Female, FGD, civil society group
Reputation, fear of rejection, and challenges finding a partner	Expectation of judgment and rejection from potential partners, friends and their families, and worry that peers would gossip about status. These fear and negative experiences added to disclosure challenges	“If sometimes I fight with someone, maybe a relative. They tell you about your status [reveal my status to others], that’s why I don’t want to tell them.” – Male, 53, key informant interview<br><br>“Right now there is this guy who is showing interest in me. I was still thinking about, if I agree to start a relationship with him, will he understand my situation? I will have to explain my situation to him. I don’t know if he will accept it or will just carry on with his life”
Disclosure challenges	Disclosing one’s HIV status can be a difficult conversation, and some participants – particularly younger participants sought to avoid it	“[Our relationship] has changed because we didn’t talk for quite some time. He stays in Durban and at the time I was in the Eastern Cape. We didn’t talk and he didn’t want me to visit him. He then decided around December that I can visit him. We never spoke about it, I used to take my treatment in front of him, I don’t know if he would hide his or what but I would take mine at the time when I had to. We are okay now, we communicate.” - Female, 26, key informant interview
**3.Understanding of U=U/TasP and its role in self-image and relationships**	Study respondents were recruited from two populations: PLHIV referred from public sector clinics and PLHIV identified through a civil society organization that works on TasP/U=U. Most participants recruited from the clinic were not aware, or were not confident, that ART leading to viral suppression prevents HIV transmission.	“Yes I’ve heard that on Facebook but they said after some time when taking your treatment you can’t transmit. But, I don’t know how true that is.”- Female, 26, key informant interview<br><br>"If you stick to your treatment you don't have to worry about infecting your partner. [Being virally suppressed,] I feel normal. I feel like anyone else. I can start a relationship with anyone I want." Female, 30, civil society group
