# Supplementary material for: “I was scared dating… who would take me with my status?”—Living with HIV in the era of UTT and U = U: A qualitative study in Johannesburg, South Africa
Source: PLOS Glob Public Health. 2023 Oct 13;3(10):e0000829. doi: 10.1371/journal.pgph.0000829 (PMC10575521; doi:10.1371/journal.pgph.0000829)
Supplement: S2 Text — (PDF) [file pgph.0000829.s003.pdf]

Study ID | | | | |

Key Informant Interview Guide

Study ID ___________________________________ Study site ___________________________________

Instructions for the interviewer

Step 1: Informed Consent and Introduction:

Ask the participant for a few minutes of their time. Introduce yourself and the study. Begin the informed consent as per the training. If consent is granted, leave the informed consent sheet with the participant.

Was verbal informed consent obtained (including consent to audio record the interview)?

YES ________ (proceed with interview)

NO ________ (STOP! Thank the participant for their time but do not proceed with the interview)

Interviewer Name and Surname _________

1. **Interview Date (DD/MM/YYYY) __________________**
2. **Time Start__________ Time Finish___________**
3. **Interviewer signature__________**

PART A. Participant Details

Name of Participant: ___________________________________

Surname of Participant: ___________________________________

Participant Date of Birth: ___________________________________

Age in years: ___________________________________

Sex: 0= Male

1= Female

Study ID | | | | |

PART B. Questionnaire

---

1. **Experience with the HIV diagnosis with partner/family/friends**

   a. Tell me about your HIV journey? When were you diagnosed?
   b. How do you feel about your HIV positive status?
   c. Have you disclosed you HIV status to anyone? If yes, who did you disclose it to?
   d. How do your family/friends feel about your diagnosis? (**Probe**: i.e. If the response is ok with it, why? What do you they mean?)
   e. Do you have a partner?
   f. How have you experienced your relationship with your past/current partner after your HIV diagnosis? (**Probe**: What do you mean? Please tell me more?)
      i. Did the HIV diagnosis affect any of your relationships?

2. **Experience with the treatment**

   a. How long have you been on HIV treatment?
   b. Can you tell me a little more about your journey with HIV treatment so far?
   c. Did you encounter any challenges with taking treatment (**Probe**: How did/do you manage?)
   d. What benefits have you experienced since starting treatment?
   e. What would you say are you most important reasons or motivations for you to continue taking treatment?

3. **Perception of treatment as prevention**

   a. Do you know you partner's HIV status?
   b. In your current/future partnership, how do you prevent your partner from getting infected?
   c. Do you feel responsible for protecting your current/current partner? How do/will you manage this responsibility and why?
   d. Have you heard the phrase 'the virus stops with me'? and what do you think it means?
   e. How - if at all - has HIV changed the way you think about sex?
   f. Have you heard that ART can prevent transmission? (**Probe**: Please tell me more about how you think treatment can prevent transmission?)

   DATA COLLECTOR - PROVIDE BRIEF INFORMATION ON U=U/TASP

   g. What role - if any - do you see for ART in preventing transmission of HIV to your current/future partners?
   h. What are your feelings about using lifelong ART to prevent transmission?
   i. What concerns do you have about using ART to prevent transmission?

4. **Comments on a communication tool**

   a. Considering what you know about the benefits of treatment, what is the best way of communicating this message to PLHIV?
   b. What do you think about a mobile application to communicate the message about treatment as prevention?
   c. Have you got some thoughts about U=U messages that should go into this application?
