# Supplementary material for: “I was scared dating… who would take me with my status?”—Living with HIV in the era of UTT and U = U: A qualitative study in Johannesburg, South Africa
Source: PLOS Glob Public Health. 2023 Oct 13;3(10):e0000829. doi: 10.1371/journal.pgph.0000829 (PMC10575521; doi:10.1371/journal.pgph.0000829)
Supplement: S3 Text — (PDF) [file pgph.0000829.s004.pdf]

***Integrating U=U into HIV Counselling in South Africa (INTUIT-SA) final: May 2021***

## FOCUS GROUP DISCUSSION

Note to facilitator:

1. Please ensure that the participants have consented to the study and signed the relevant form.
2. If everyone is willing to participate and be recorded, then put on the digital recorder, speak and say:

Name of facilitator:
Date of session:
State which group you will be facilitating i.e. men/ women/ couples:

## FOCUS GROUP DISCUSSION GUIDE

### Introductions

Ask the participants to introduce themselves, say how long it is since they discovered they were HIV positive, say how many years since they started treatment, and something about themself.

1. Could you please describe your journey since learning your HIV positive status??

### Questions about HIV treatment motivations

2. What first motivated you to seek treatment?
3. We are going to talk about challenges and benefits of taking treatment. Why is taking treatment important to you? When does taking treatment become less important? What motivates you to take your pills and to go to the clinic for refills, check-ups and lab tests? What demotivates you?
    a. What benefits have you experienced since starting treatment?
    b. What challenges have you faced in accessing treatment? How have you overcome these challenges? (Facilitator to probe about issues such as awareness of treatment options, access to treatment, and issues like stigma etc.)
    c. What challenges have you faced in taking treatment? How have you overcome these challenges? (Facilitator to probe about the influence of treatment's effects on the body as well as other challenges for taking treatment).

### Questions about treatment, prevention, and sexual partnerships

1. How has HIV affected your sexual relationships?
    a. Is your partner aware of your HIV status?
    b. Do you feel responsible for protecting your current or future partner? Why or why not?
    c. How do you prevent your partner from becoming infected?

   d. Knowing you live with HIV, how does that make you feel about whether you will transmit HIV and how you can protect others from HIV?
   e. If you are not currently in a relationship, how does living with HIV affect your ability to find new partners (if at all)?

2. Have you heard that Antiretroviral Therapy (ART) can prevent transmission?
   a. For those of you who have heard about it, how and when was the first time you heard about treatment as prevention?
   b. How do you think treatment prevents transmission?
   c. Are you aware of the term U=U? What do you understand by this term?

3. Have you ever had a viral load test?
   a. Did you understand what the viral load test was for? Please explain what it is for?
   b. How is viral load linked to prevention?
   c. Did your viral load result change the way you felt about your status?
   d. What did you learn from the counsellor about how to interpret your viral load result?

4. Thinking about your own life, Do you have any concerns about using ART to prevent transmission?

## Questions about communication products

We are in the process of developing some communication materials to encourage other people living with HIV to use Treatment as Prevention.

1. Do you think that encouraging ART as prevention is a good message to share with people living with HIV (PLHIV)? Why or why not?
2. How would you encourage others to seek treatment? What assists people to seek treatment?
3. How would you inform others of the importance of taking their treatment? What assists people to take their treatment? What assists people to take their treatment all the time?
4. What do you think are useful ways to communicate this message? Why and why not?

Is there anything else you think people should know about treatment as prevention?

**Thank you for sharing with us.**
**Do you have any questions for me?**
