# Supplementary material for: “I was scared dating… who would take me with my status?”—Living with HIV in the era of UTT and U = U: A qualitative study in Johannesburg, South Africa
Source: PLOS Glob Public Health. 2023 Oct 13;3(10):e0000829. doi: 10.1371/journal.pgph.0000829 (PMC10575521; doi:10.1371/journal.pgph.0000829)
Supplement: Attachment — Submitted filename: Response to reviewers, 6jun2023.doc [file pgph.0000829.s005.doc]

**Response to reviewers:**

**Reviewer #1:** 1. The IDIs and FGDs had mutually exclusive participants or there was an overlap? Please clarify.

The IDIs and FGDs were mutually exclusive. We further conducted follow-up interviews with a subset of the participants (n=29) to get an in-depth understanding of their journeys of living with HIV.

Page 2: Line 32-34 “Follow-up interviews were conducted with 29 IDI and FGD participants, to gain a deeper understanding of their journey living with HIV”

2. Since this is framed around UTT, can authors include how experiences changed before and after UTT? I am not able to see the utility of UTT otherwise.

We thank you for your comment. We do not have data from the pre-UTT era, however there is a wealth of prior studies that look at the meaning ascribed to HIV and experiences of PLHIV in the pre-UTT era. We believe that UTT remains a useful reference point as it marks the current era in which – due to universal ART eligibility – people can be diagnosed with HIV, start treatment, and live a long life, *without ever getting sick.* This is a factor in the “normalization” of HIV as a chronic disease like any other. Additionally, UTT also marks the era in which ART can be used by all PLHIV, regardless of disease stage, to prevent onward transmission. Indeed, treatment as prevention one of the main rationales for the UTT policy. The scientific evidence on treatment as prevention is now clear: “undetectable” HIV is “untransmittable”, i.e. U=U. This information has just started to penetrate South Africa, and forms the context for the study. For these important reasons, UTT shapes the context of the data collected.

We have changed the title to: (p1)

**“I was scared dating… who would take me with my status?”- Living with HIV in the era of UTT and U=U: a qualitative study in Johannesburg, South Africa”**

We have edited the abstract to emphasize the rationale: (p2, line 26)

“South Africa rolled out Universal Test-and-Treat (UTT) in 2016, extending treatment eligibility to all persons living with HIV (PLHIV) to reduce HIV transmission and improve the health of PLHIV. We sought to understand how PLHIV in Johannesburg, South Africa, interpret and experience their HIV status, five years into the UTT era.”

We have added a paragraph to the discussion section that contextualizes our findings within prior work on experiences and meaning of HIV in the pre-UTT era as well as studies on the impact of UTT on stigma.

Pages 20: lines 504 - 515, “The scale-up of ART has transformed HIV into a manageable chronic condition. Universal eligibility under UTT means that PLHIV do not have to wait to start therapy and that PLHIV have access to ART as a means to prevent onwards transmission. Still, while participants in our study viewed HIV as “normal” and “common”, we found that internalised stigma remained pervasive. Our findings echo those of other studies that have found that stigma, though reduced[36], remains high despite the scale-up of treatment[30, 32]. Similarly, recent findings showed little evidence that UTT substantially reduced HIV stigma among PLHIV, community members, and health workers (HCWs) not living with HIV [31]. Our results are particularly concerning as studies have shown that stigma is associated with depression among PLHIV and negatively impacts quality of life [38, 39]. Additionally, stigma is a barrier to HIV testing and ART adherence, and, thus undermines efforts towards the elimination of HIV[37, 40].”

3. “Despite the normalization of HIV as a chronic disease” is very loaded since normalization can mean many different things. May consider changing it and making it more specific.

We thank you for your comment and have rephrased. We note that the word “normal” is taken directly from a quote of a participant.

Page 10: line 255, “Despite the ability to live a “normal” life with HIV, participants still reported feelings of guilt and shame related to their HIV diagnosis.”

4. ‘Participants did not view HIV as a death sentence with treatment widely available’ – this is repetition of previous line in different words. May consider removing since it doesn’t add any value.

Page 10: line 248, The line “Participants did not view HIV as a death sentence with treatment widely available” has been removed.

5. The paragraph following ‘1.2. Persistent HIV stigma is linked to sexual transmission’ doesn’t have material related to this specific heading.

Good catch. That was an error. We have revised the paragraph so that it follows from the heading and matches the data. We now write:

Page 11: line 282 -290 “Despite the waning association of HIV with death, HIV still remained highly stigmatised as a sexually transmitted disease. Stigma is a “mark of disgrace” and its link with transmission is twofold. First, HIV is stigmatized because it is linked to stigmatized sexual behaviour (e.g. promiscuity, infidelity) and thus inherits the stigma associated with that behaviour. Second, because HIV is stigmatized, sexual transmission of the virus involves giving an undesired “mark of disgrace” to an intimate partner. It is unsurprising, therefore, that many participants viewed HIV transmission as a moral transgression.”

**Reviewer #2:**

1. The numbers do not add up in the result section of the abstract. Kindly review.

We thank you for your comment. We invited a subset of the participants for follow-up interviews to get a full understanding of their journeys of living with HIV.

Page 2: Line 32-34, “Follow-up interviews were conducted with 29 IDI and FGD participants, to gain a deeper understanding of their journey living with HIV.”

2. Don't you think the clinic setting had an influence on the responses for the in-depth interviews?

We thank you for your comment. We recognise the clinic setting as a limitation.

Page 20: lines 566-570, “Similarly, the participants recruited for IDIs were in care and knowledgeable of benefits of ART through experience and during counselling. Responses might have been different if the participants were not engaged in clinical care and recruited from clinical settings.”

3. Although this study is said to be part of a larger one which has its objectives, how were the domains/ themes objectively identified/ determined for the qualitative study? what was the methodological orientation or theory

We thank you for your comment. We have clarified:

Page 7: Lines 189 -193, “An inductive approach (S1 Table. Codebook), as used in grounded theory, was used to derive emergent themes from our data. Since the themes were data driven, they did not mirror the exact questions asked of participants and rather sought to capture the “deep stor(ies)” of our informants [35]. As emergent themes were identified and refined, new codes were developed and transcripts revisited to identify subthemes and links between these constructs.”

4. Since you reported purposive sampling (though not very clearly done), what guided participant selection?

Thank you for the opportunity to clarify our sampling strategy. We have now revised:

Pages 5 – 6 : Lines 130 -165, “Participant selection was guided by two goals. First, we sought to recruit a sample of adult PLHIV that were diverse in terms of gender, age, and duration on treatment, and who accessed care at public facilities. Second, we sought to recruit participants with different levels of knowledge about U=U, to better understand whether the knowledge of U=U shaped people’s experience of living with HIV.

To achieve these goals, we started by recruiting PLHIV at a primary health clinic immediately after HIV diagnosis or counselling. Our recruitment focused on participants who were either ART naïve or had been on treatment for less than six months – to better capture the experiences of people coming to terms with their HIV status in the UTT era. Potential participants were referred by lay HIV counsellors and introduced to the study staff. Those who agreed to be part of the study participated in an in-depth interview, an appropriate method of data collection given privacy concerns for people who may still be accepting their HIV status. We excluded patients who were unable or too sick to participate, unwilling to provide consent, or planned to get treatment elsewhere. Additionally, women who were pregnant at HIV diagnosis were excluded because treatment and care goals differ for pregnant women.

All participants recruited from clinical settings were unaware of U=U, which we expected as U=U had not yet been integrated into HIV counselling. We therefore engaged in a second recruitment strategy, obtaining participants through a civil society organization that seeks to educate people on U=U. We used snowball sampling, starting with a few initial contacts, in order to recruit participants for three focus group discussions (FGDs), which we stratified by gender were conducted using an FGD guide (S3 text. FGD Guide). The FGDs allowed us to get multiple perspectives on the same theme and enabled us to have a discussion about U=U in which participants could hear other participants endorsing the science of U=U. These FGDs were stratified by gender to promote open dialogue among participants around topics involving sexual relationships. A third (n=6) FGD consisted of younger (**Table 1**) participants.

Did you consider duration on treatment in selecting potential respondents, since this has been shown to influence stigma-related responses and client knowledge of their condition.

Duration of treatment was taken into consideration. We recruited and enrolled patients who had years of experience living with HIV and being on ART but we also included patients who were ART naïve and recently diagnosed. We now write:

Page 5: Lines 130 - 134 “Participant selection was guided by two goals. First, we sought to recruit a sample of adult PLHIV that were diverse in terms of gender, age, and duration on treatment, and who accessed care at public facilities. Second, we sought to recruit participants with different levels of knowledge about U=U, to better understand whether the knowledge of U=U shaped people’s experience of living with HIV.”

5. How objective was the data analysis? sharing the coding tree, as prescribed by Consolidated criteria for reporting qualitative research (COREQ) would be helpful.

We thank you for your comment, we have now included a COREQ checklist (S1_text. COREQ Checklist) and codebook (S1_table. Codebook) and have added the following to the methods section:

Page 7: Lines 185-192, “Each transcript was coded by two coders independently, and the coders held detailed discussions to reconcile differences in codes and interpretation. In cases where the difference could not be reconciled, a third member of the research team was assigned to help resolve the differences.

An inductive approach (S1 Table. Codebook), as used in grounded theory, was used to derive emergent themes from our data. Since the themes were data driven, they did not mirror the exact questions asked of participants and rather sought to capture the “deep stor(ies)” of our informants [35]. As emergent themes were identified and refined, new codes were developed and transcripts revisited to identify subthemes and links between these constructs.”

6. Who did the interviewing? what was their relationship with the respondents? what is their qualification?

The interviewer was well trained and experienced in both qualitative research. The interviewer had no relationship with any of the participants. The interviewer is a qualified study coordinator.

We now write in the Methods section:

Page 5: Lines 123-126 “Dedicated study staff with training and experience in qualitative interviews conducted all screening, consent procedures, and data collection.”

7. The quotation between 57 and 62 is not very clear. Could you kindly look at it again?

We thank you for this comment, we have streamlined the quote to make it more clear:

Pages 11: Lines 292-298 *"I started treatment in 2017 because I was in denial, was hiding it even at home, this thing, and I was scared to talk about this situation….  Unfortunately at the time I broke my virginity I got pregnant and I got HIV and it was so sad to me that I had disciplined myself for such a long time.” –* ***Female, FGD, civil society group*** *.*

8. The quotation in 134-136 don't seem to support the statement in 131. Kindly clarify

We thank you for you pointing out this error. The quote is unrelated because both partners are HIV positive. The other partner might be concerned about acquiring resistance since they do not trust that their partner is taking treatment regularly. We have removed the quote below:

Pages 14: Lines 370-372 *“He does understand but sometimes it becomes a challenge because his work requires him to travel and I am not sure if he complies with his treatment therefore I prefer for us to always use protection.”-* ***1013*** ***female, 23, key informant interview***

9. Did clients validate the findings?

Due to time constraints, we were not able to share the findings with the participants. However, during data coding we reached saturation, meaning the same themes were common across participants. We also presented the results and obtained feedback from our study’s stakeholder advisory board which includes PLHIV.

10 Can you provide information on those who refused to take part in the study?

We thank you for your comment. Although this information is not documented, the majority of participants who were referred agreed to participate. The few who were not enrolled did not have enough time to wait while the interviewer was busy with other interviews.

11. Although you said you tried to balance the genders in selecting respondents, the quotations were largely from females. Does this not affect the rigor of your findings?

We thank you for your comment. Our participants were predominantly female. hence majority of the quotes were from female participants. We have added this as a limitation. We now write:

Page 21: Lines 577-580,“Despite conducting an all-male focus group, a limitation of the study is the small number of interviews conducted with recently-diagnosed, younger male respondents. Results may not be generalizable to this population.”

12. Table 1 shows that you had 21 participants for the first FGD. How were they managed to ensure that contributions were received from all participants. How was data collected during the focus group discussions? was it also video taped or were field notes taken?

We thank you for your comment. The first two FGDs were split by gender and conducted separately to encourage participation.

We now write in the Methods section:

Pages 6-7: Lines 171-177, “The facilitator encouraged participation and ensured that every participant was given an opportunity to contribute to the discussions. A note taker carefully observed dynamics between the participants. The sessions were audio recorded and field notes were taken. The audio recording was transcribed verbatim and translated.”

13. The text mentions a third FGD but table 1 has only two. Kindly look at that again.

Thank you. that was an oversight.

Table 1, on page 7 has been amended to include all 3 FGDs.

14. How was the data collected in Sotho and Zulu languages managed well before analysis?

Thank you for the opportunity to clarify.

We now write in the Methods section:

Page 7: Lines 182-188, “Interviews and focus group discussions were audio-recorded, transcribed verbatim, translated to English by a well-recognised company for transcription and translation from both Sotho and Zulu to English and analysed thematically. The original transcripts were compared against the translated by a member of research team to ensure validity. Each transcript was coded by two coders independently, the coders held detailed discussions to reconcile differences in codes and interpretation. In cases where the difference could not be reconciled, a third member of the research team was assigned to help resolve the differences."

**Reviewer #3**: This manuscript presents qualitative data concerning PLWHA in South Africa's experiences with stigma and relationships in the era of UTT and TasP. The manuscript presents interesting findings, with potential implications for patient counselling and support, but there are several areas where the manuscript could be strengthened.

Minor comments:

The data availability statement is a little vague, and it isn’t clear to me whether it is feasible or ethical to make the qualitative datasets available, without explicit prior permission from the participants.

We thank you for your comment, appropriate data generated or analysed during the study can be made available upon reasonable request.

I would check the keywords and spell out some of them (especially Treatment as Prevention rather than TasP), and consider adding “qualitative methods” as a keyword.

We thank you for your comment.

Page 1: “qualitative methods” added as a key word.

TasP changed to Treatment-as-Prevention. HIV, ART, and Stigma added to the list of key words.

Introduction:

Would include where South Africa is at with their 90-90-90/95-95-95 goals

We thank you for your comment. We have now added:

Pages 3-4: line 84 -87: “In 2020, 94% of South African PLHIV knew their HIV status, of which 74% were on treatment, of which 67% were virally suppressed [1].”

The introduction covers a lot of ground, but it isn’t clear what gaps this study intends to fill. It seems like the goal of the study is to understand how UTT and TasP impacts stigma and people’s lives(?), but this needs to be more clearly stated, and additional literature provided. The PopART study may be a useful place to look, as they’ve done a fair amount of work on stigma in the context of promoting TasP.

We have now clarified the gap the study is trying to fill by stating our study question explicitly at the start of the paper:

“How do people living with HIV (PLHIV) today interpret and experience their HIV status?”

The goal of the study is to describe how people make meaning of their HIV status in the current era – an era is defined by widespread access and universal eligibility for treatment as well as growing awareness that treatment = prevention. This is a descriptive, qualitative paper and we did not set out to draw conclusions about causal impact of UTT or TasP. Rather these concepts shape the context in which PLHIV are interpreting and experiencing their HIV status today. While persistent stigma linked to transmission turned out to be a key finding of the study, we were agnostic about what we would find (consistent with our grounded theory approach) and did not ask people to report on stigma specifically. The paper thus did not set out to evaluate the impact of UTT and TasP on stigma, but rather to characterize how PLHIV understand their status and the role it plays in their lives. As we describe in the introduction, ART implies that PLHIV can have a normal life expectancy. But does this mean that they can have a “normal” life? As we find out in this study, the answer is both yes and no.

We have added relevant references on PopART and other papers that have looked at stigma in the context of TasP. We discuss these in the discussion section: (p18, lines 504-513)

“The scale-up of ART has transformed HIV into a manageable chronic condition. Universal eligibility under UTT means that PLHIV do not have to wait to start therapy and that PLHIV have access to ART as a means to prevent onwards transmission. Still, while participants in our study viewed HIV as “normal” and “common”, we found that internalised stigma remained pervasive. Our findings echo those of other studies that have found that stigma, though reduced[36], remains high despite the scale-up of treatment[30, 32]. Similarly, recent findings showed little evidence that UTT substantially reduced HIV stigma among PLHIV, community members, and health workers (HCWs) not living with HIV [31]. Our results are particularly concerning as studies have shown that stigma is associated with depression among PLHIV and negatively impacts quality of life [38, 39].”

The rationale for doing both in-depth interviews and FGDs is unclear, especially since the topics covered seem to be the same.

We thank you for your comment. We now provide the rationale:

Page 5, Lines 136-141, “Our recruitment focused on participants who were either ART naïve or had been on treatment for less than six months – to better capture the experiences of people coming to terms with their HIV status in the UTT era. Potential participants were referred by lay HIV counsellors and introduced to the study staff. Those who agreed to be part of the study participated in an in-depth interview, an appropriate method of data collection given privacy concerns for people who may still be accepting their HIV status.

Page 6, Lines 153-165, “All participants recruited from clinical settings were unaware of U=U, which we expected as U=U had not yet been integrated into HIV counselling. We therefore engaged in a second recruitment strategy, obtaining participants through a civil society organization that seeks to educate people on U=U. We used snowball sampling, starting with a few initial contacts, in order to recruit participants for three focus group discussions, which we stratified by gender. By virtue of the sampling strategy, these participants had been living with HIV for longer and most were aware of U=U. The FGDs allowed us to get multiple perspectives on the same theme and enabled us to have a discussion about U=U in which participants could hear other participants endorsing the science of U=U.”

The sampling frame and inclusion criteria for the in-depth interviews is also rather vague.

We thank you for the opportunity to clarify. All participants were enrolled in the study by trained interviewers via referral from PHC-based lay HIV counsellors, immediately after HIV diagnosis or counselling. Participants were interviewed on the day of enrolment into the study. Participants self-reported being newly diagnosed or diagnosed less than six months from the date of enrollment during the screening process. We excluded patients who were unable or too sick to participate, unwilling to provide consent or planned to get treatment elsewhere. Additionally, women who were pregnant at HIV diagnosis were excluded from the study because in-pregnancy treatment initiation and care processes differ from that of non-pregnant women.

We now write:

Page 5: Lines 130-144, "Participant selection was guided by two goals. First, we sought to recruit a sample of adult PLHIV that were diverse in terms of gender, age, and duration on treatment, and who accessed care at public facilities. Second, we sought to recruit participants with different levels of knowledge about U=U, to better understand whether the knowledge of U=U shaped people’s experience of living with HIV.

“To achieve these goals, we started by recruiting PLHIV at a primary health clinic immediately after HIV diagnosis or counselling. Our recruitment focused on participants who were either ART naïve or had been on treatment for less than six months – to better capture the experiences of people coming to terms with their HIV status in the UTT era. Potential participants were referred by lay HIV counsellors and introduced to the study staff. Those who agreed to be part of the study participated in an in-depth interview, an appropriate method of data collection given privacy concerns for people who may still be accepting their HIV status. We excluded patients who were unable or too sick to participate, unwilling to provide consent, or planned to get treatment elsewhere. Additionally, women who were pregnant at HIV diagnosis were excluded because treatment and care goals differ for pregnant women.

Page 6: Lines 153-165, “All participants recruited from clinical settings were unaware of U=U, which we expected as U=U had not yet been integrated into HIV counselling. We therefore engaged in a second recruitment strategy, obtaining participants through a civil society organization that seeks to educate people on U=U. We used snowball sampling, starting with a few initial contacts, in order to recruit participants for three focus group discussions, which we stratified by gender. The FGDs allowed us to get multiple perspectives on the same theme and enabled us to have a discussion about U=U in which participants could hear other participants endorsing the science of U=U.”

The thematic analysis approach needs to be a little clearer, and it would be helpful if the authors provided citations to support their analytical approach.

We thank you for your comment. We have now clarified:

Page 7: Lines 189 -193, “An inductive approach (S1 Table. Codebook), as used in grounded theory, was used to derive emergent themes from our data. Since the themes were data driven, they did not mirror the exact questions asked of participants and rather sought to capture the “deep stor(ies)” of our informants [35]. As emergent themes were identified and refined, new codes were developed and transcripts revisited to identify subthemes and links between these constructs.”

The coding process is also missing some details, including how many coders there were and their backgrounds/positionality. Re-working the methods in line with the COREQ checklist would help clarify a lot of the questions.

We have added the COREQ checklist as supplemental material “S1 text. COREQ checklist” and now mention it with a callout in the Methods section. Additionally, we now write:

P7, lines 184-188: “The original transcripts were compared against the translated by a member of research team to ensure validity. Each transcript was coded by two coders independently, and the coders held detailed discussions to reconcile differences in codes and interpretation. In cases where the difference could not be reconciled, a third member of the research team was assigned to help resolve the differences.”

Including the interview and FGD guides, as well as the final codebook as supplemental materials would be helpful.

We have added the codebook and IDI and FGD guides as supplemental materials “S2 text. IDI Guide”, “S3 text. FGD Guide”, and “S4 text. Codebook”, and we include callouts in the text.

Page 5: line 128 “Interviews were conducted using an IDI guide (S2 IDI Guide)”

Page 6: Lines 160-162, we used snowball sampling, starting with a few initial contacts, in order to recruit participants for three focus group discussions (FGDs), which we stratified by gender were conducted using an FGD guide (S3 text. FGD Guide).

Page 7: Lines 189-193, An inductive approach (S4 Table. codebook), as used in grounded theory, was used to derive themes from our data. Since the themes were data driven, they did not mirror the exact questions asked of participants and they are not necessarily reflective of our own interests on the subject as shown in other research [35] Additional codes were added as themes emerged.

Results

The results would benefit from an introductory paragraph mapping out the themes and sub-themes to be described.

We thank you for your comment, we have added an introductory paragraph that shows the themes and sub-themes included in the results section

Page 9: Lines 230 -239, “The IDIs and FGDs yielded the following emergent themes. First, while PLHIV viewed HIV as a common chronic condition, they still perceived stigma associated with HIV, engaged the language of blame in talking about HIV transmission, and many had low self-image associated with being HIV-positive. Second, PLHIV experienced transmission anxiety, i.e. the fear of passing HIV on to someone else; several reported fears of future rejection by romantic partners, disincentivizing disclosure, and many questioned whether they would be able to have sexual relationships or a family. Third, most participants recruited from the clinic were not aware, or were not confident, that ART leading to viral suppression prevents HIV transmission; however, those respondents where were aware of U=U/TasP had less anxiety about HIV transmission and had more positive self-image.”

The ID numbers attached to the quotes are not necessary, since they do not mean much to the reader. I would either drop or replace with something more meaningful (e.g., demographic information like age, time since diagnosis, other).

We thank you for your comment. All ID numbers have been removed.

I think making the manuscript objectives and guiding framework(s) clearer in the introduction would make the organization and flow of the results a little smoother.

We thank you for your comment, We have added text that clarifies the main objectives and guiding framework.

Pages 4: Lines 184, “In this qualitative study, we sought to understand how PLHIV in Johannesburg, South Africa, experience and interpret their HIV status in the UTT era; how being HIV-positive affects their lives; and what role, if any, knowledge of TasP plays in their experience of living with HIV. Understanding the meaning ascribed to HIV and the challenges PLHIV face due to their status is critical to support psychosocial well-being and to motivate testing and ART adherence.”

Discussion

The lack of male participants in the qualitative sample also seems like a potential limitation. I’m curious as to why there wasn’t more of an effort to balance the sample, but maybe this wasn’t important for the research questions? Would be interesting to know the extent to which participants concerns are gendered, especially given all the concern women expressed about future reproductive health, pregnancies.

We thank you for your comment. Challenges recruiting younger male participants from the clinic due to the fact that men do not generally seek help until they are seriously ill led us to conduct a Focus Group of entirely male participants reached through snowball sampling. Nevertheless, the paucity of IDIs with recently-diagnosed men is a limitation of the study.

We have added to the limitations:

Page 21: line 577-580.,“Despite conducting an all-male focus group, a limitation of the study is the small number of interviews conducted with recently-diagnosed, younger male respondents. Results may not be generalizable to this population.”

Given how large the literature on HIV stigma is, and the growing literature on UTT and TasP, the discussion could do a little more in term of linking findings to this larger literature, as well as making recommendations.

We have added relevant literature to the discussion and conclusion. We now write:

Page 18-19: line 504-522. ”The scale-up of ART has transformed HIV into a manageable chronic condition. Universal eligibility under UTT means that PLHIV do not have to wait to start therapy and that PLHIV have access to ART as a means to prevent onwards transmission. Still, while participants in our study viewed HIV as “normal” and “common”, we found that internalised stigma remained pervasive. Our findings echo those of other studies that have found that stigma, though reduced[36], remains high despite the scale-up of treatment[30, 32]. Similarly, recent findings showed little evidence that UTT substantially reduced HIV stigma among PLHIV, community members, and health workers (HCWs) not living with HIV [31]. Our results are particularly concerning as studies have shown that stigma is associated with depression among PLHIV and negatively impacts quality of life [38, 39]. Additionally, stigma is a barrier to HIV testing and ART adherence, and, thus undermines efforts towards the elimination of HIV[37, 40]. HIV stigma is often internalized, leading to continued psychosocial harm[7]. Our findings show that study respondents reported feelings of shame and guilt for contracting the virus and perceived their infection as a moral transgression. These findings are consistent with prior research in sub-Saharan Africa, which showed that HIV infection was perceived to be a result of risky sexual behaviour[3, 8].

We also added a recommendation in the conclusion,

Page 21: line 587-588 ‘’Our findings suggest that expanding ART eligibility alone is not enough to address the challenges. Disseminating information on TasP/U=U could reduce the psychosocial burdens of HIV including self/internalized stigma, encourage disclosure, and remove barriers to HIV testing and treatment adherence.”
